# A Case Control Study Examining the Patterns and Predictors of Referral to Cancer Rehabilitation at Canada's Largest Comprehensive Cancer Centre

**DOI:** 10.1002/cam4.71046

**Published:** 2025-07-10

**Authors:** Jennifer M. Jones, Rogih Andrawes, Michelle A. Weller, Adrienne Lam, Gilla K. Shapiro, Madeline Li, Danielle Rodin, Lisa Avery

**Affiliations:** ^1^ Department of Supportive Care Princess Margaret Cancer Centre Toronto Ontario Canada; ^2^ Department of Psychiatry, Temerty Faculty of Medicine University of Toronto Toronto Ontario Canada; ^3^ Temerty Faculty of Medicine, Institute of Medical Sciences University of Toronto Toronto Ontario Canada; ^4^ Royal College of Surgeons Dublin Dublin Ireland; ^5^ Department of Radiation Oncology, Temerty Faculty of Medicine University of Toronto Toronto Ontario Canada; ^6^ Department of Biostatistics Princess Margaret Cancer Centre Toronto Ontario Canada

## Abstract

**Background:**

Cancer rehabilitation has become increasingly relevant as the number of cancer survivors grows, coupled with the high‐documented rates of adverse effects and related disability. Cancer rehabilitation can reduce functional limitations among cancer survivors and enhance their well‐being. However, only a small proportion of individuals are referred to rehabilitation services. To identify and address disparities and foster access, it is essential to develop a better understanding of the factors that drive referral to cancer rehabilitation services.

**Methods:**

The purpose of this study was to: (1) describe the sociodemographic and clinical characteristics and symptom burden of patients who were referred to the Princess Margaret Cancer Rehabilitation and Survivorship (CRS) Program between 2017 and 2019 and (2) Compare these variables between patients who were referred to CRS (*n* = 2783) and matched cases who were not referred over this period (*n* = 18,434). A retrospective secondary analysis of data extracted from the Princess Margaret (PM) Cancer Registry, electronic patient records, and patient‐reported outcome data (PROMs) (including ESAS‐r and ECOG status) was performed. Summary statistics were used to describe the patients referred to the CRS program. Multivariable logistic regression modelling was used to identify factors associated with likelihood of referral.

**Results:**

Most referred patients were female (74%), English speakers (93%) and half lived within 15 km of the referred hospital. The most common reasons for referral were musculoskeletal impairment (26%) and lymphedema (25.4%). Many patients (45%) had multiple reasons for referral. Several key predictors of referral were identified including closer distance to hospital, lower age (< 65 years), cancer site, and completion of PROMs. For those who completed PROMs, patient reported function status and pain scores were related to referral.

**Conclusion:**

The findings can be helpful in optimizing the referral processes and addressing disparities regarding access to cancer rehabilitation. Solutions are likely multifaceted including health care provider and patient education and systemic changes to address barriers.

## 1 Background

There has been a rapid increase in the number of individuals with a history of cancer diagnosis since the 1980s [1]. This can be attributed to several factors, including a growing and aging population, as well as advancements in cancer control, early detection methods, and the development of more efficient treatments [1]. It is anticipated that this trend will continue, with an additional projected rise of 30%–40% individuals living with a history of cancer by 2040 [2].

Cancer survivors encounter a range of physical, functional, and psychosocial difficulties during and after their cancer treatment, which frequently go undetected and receive insufficient attention [3, 4, 5, 6, 7, 8]. These challenges can significantly impact their quality of life [9] and hinder one's ability to fully engage in work and life roles [3, 7, 10, 11, 12, 13, 14, 15]. These effects vary depending on treatment exposure and individual patient factors [16] and are known to be higher in medically underserved populations [17]. This presents both a challenge and an opportunity to better meet the comprehensive healthcare needs of all cancer survivors [18, 19, 20, 21].

Comprehensive cancer rehabilitation focuses on the prevention and treatment of immediate, persistent, or late effects of cancer and cancer treatment, and the maintenance of health [22, 23, 24, 25, 26]. This has become increasingly relevant as the number of cancer survivors grows, coupled with the documented high rates of physical impairment and disability [27, 28, 29]. Supported by this robust body of evidence [22, 23, 24, 30, 31] and an increasing number of guidelines providing specific recommendations for cancer rehabilitation, there is a growing consensus on the need to integrate rehabilitation as a vital component of cancer care [32, 33, 34, 35]. However, despite the strong evidence base and growing demand for cancer rehabilitation services, current evidence suggests that only a small proportion of individuals are referred to rehabilitation services to address adverse cancer‐related side effects and associated disability [11, 36, 37, 38, 39]. Further, the scarcity of healthcare resources, including both financial and human resources, poses a limitation to the capacity of supportive care services including rehabilitation, making it crucial to identify which patients are in the greatest need of a referral and ensuring equal opportunity for all who need access [21, 40].

In order to identify and address disparities and foster access to cancer rehabilitation services, it is essential to develop a better understanding of the factors that are associated with referral to cancer rehabilitation services. To date, a small number of studies have focused on assessing the disparities in patient referrals to cancer rehabilitation services. Research conducted in Denmark, where cancer rehabilitation is widely available and free of charge by referral, has found that social determinants of health (SDOH), including distance from hospital, education level, socio‐economic status, and sex, have an impact on referral and attendance to cancer rehabilitation services [18, 36, 37, 38]. However, the influence of SDOH and other factors affecting access to cancer survivorship in the Canadian context, where cancer rehabilitation is less available, has not been evaluated. Furthermore, the relationship between SDOH, clinical factors, and patient‐reported symptom burden and function has yet to be examined in a combined analysis.

The purpose of this study is to examine factors that are associated with referral to cancer rehabilitation in Canada's largest cancer centre. This information will help to develop specific strategies to improve access to supportive care services. The specific objectives of this study were to: (1) Describe the sociodemographic and clinical characteristics and symptom burden of patients who were referred to the Cancer Rehabilitation and Survivorship (CRS) Program at Princess Margaret (PM) Cancer Centre between 2017 and 2019 and (2) Compare these variables between patients who were referred to CRS and matched cases who were not referred over this period.

## 2 Methods

### 2.1 Study Design

This was a retrospective case–control study of patients diagnosed with solid cancers and hematological malignancies at PM. Cases were patients referred to the PM CRS program between 1 January 2017 and 31 December 2019. This timeframe was selected as it did not overlap with COVID‐19 disruptions and to account for the 6–12 month lag in data extraction to the PM Cancer Registry. Controls were all never‐referred patients identified through the PM Cancer Registry and matched based on diagnosis month/year and cancer type. This study was approved by the University Health Network Research Ethics Board (#21‐5848).

### 2.2 Program Description

The PM CRS Program is a consultative impairment‐driven program delivered by a multidisciplinary rehabilitation team. Patients who have been diagnosed with cancer, received some part of their treatment at PM, and have identified cancer‐related impairments are eligible to be referred to the CRS program. Referrals must be made by a physician (i.e., oncologist, family doctor) or physician assistant, and self‐referrals are not accepted. All eligible referred patients undergo an initial comprehensive cancer rehabilitation assessment, and a personalized care plan is then developed based on their identified impairment(s), level of disability, and personal goals. Care within the program may include further one‐on‐one follow‐up appointments to address specific issues (e.g., lymphedema, deconditioning, fatigue, musculoskeletal issues, neurocognitive changes, difficulty with return to work) and/or group education (e.g., diet/nutrition, fear of cancer recurrence, lymphedema, brain fog). Patients can also be referred to the Cancer Rehabilitation and Exercise (CaRE) program, an 8‐week structured multidimensional program consisting of exercise and self‐management skills education delivered in‐person in a group format (CaRE@ELLICSR) [41] or a home‐based model (CaRE@Home) [42].

### 2.3 Data Sources and Variables

This study is a retrospective secondary analysis from three PM data sources including the PM Cancer Registry, routinely collected patient reported outcome measures (PROMs), and the University Health Network (UHN) Electronic Patient Record (EPR). In addition, postal code data was used to estimate distance to PM and to map patients to the Ontario Marginalization Index (ON‐Marg) and income quintile as defined by the Canadian census.

1. PM Cancer Registry: Data extracted from the PM Cancer Registry included sociodemographic information including date of birth, sex, and postal code. Clinical variables included date of diagnosis, primary cancer site, and treatments received. *Distances from PM* were estimated using the Google Driving Distance macro (Google LLC, Menlo Park, CA) and the postal code of the patients' primary residences. *Neighborhood‐level socioeconomic data*, including income and marginalization data, were obtained by first linking respondents' postal codes to data access units through the Postal Code Conversion File Plus (PCCF+) Version 8A [43]. Income data was obtained from the PCCF+ and this provided neighborhood income quintile relative to the income of the census metropolitan area from the 2021 Canadian census. In addition, we linked postal codes to the Ontario Marginalization Index (On‐Marg), an area‐specific index that highlights differences in marginalization across Ontario [44]. On‐Marg comprises four dimensions, each considered a separate index: (1) Household and Dwellings: Reflects family and neighborhood stability, types and density of residential accommodations, and family structure characteristics. (2) Material Resources: Indicates the inability of individuals and communities to access basic needs. (3) Age and Labor Force: Measures the concentration of people without employment income. (4) Racialized and Newcomer Populations: Shows the proportions of recent immigrants and visible minorities. Each dimension is standardized for each area and can be reported by quintiles, with lower quintiles representing least marginalized and higher quintiles most marginalized [44]. Based on preliminary models and to improve model interpretability, each of these On‐Marg dimensions was dichotomized prior to analysis (see Supplemental File 1).
2. Patient reported outcome measures*:* Beginning in 2013, patients at PM were asked to complete routine electronic screening with patient‐reported outcome measures (PROMs) before every outpatient visit [45]. This includes the following validated core measures: (i) the Edmonton Symptom Assessment Scale‐revised (ESAS‐r) [46] which measures common cancer symptoms including pain, tiredness, drowsiness, nausea, lack of appetite, shortness of breath, depression, anxiety, and well‐being on a 0–10 scale where 0 indicates best health and 10 worst. These were dichotomised as follows: scores of 0–3 were considered none/mild and scores of 4–10 were considered moderate/severe [46] and (ii) the Eastern Cooperative Oncology Group Patient Reported Functional Status measure (ECOG‐PRFS) [47, 48] which assesses activities and function on a 5‐point scale, where 0 is “normal with no limitations,” 1 is “not my normal self, but able to be up and about with fairly normal activities,” 2 is “not feeling up to most things, but in bed or chair less than half of the day,” 3 is “able to do little activity and spend most of the day in bed or chair,” and 4 is “pretty much bed ridden, rarely out of bed.” At appointment check in, patients were directed to complete their PROMs on tablets in the waiting areas of clinics. PROMs patient and clinician reports were then printed in the clinic, with the latter transmitted directly to the patients' electronic medical record. Patients who complete the PROMs are asked to provide consent for permission to use the patient's non‐identifying PROMs data for research purposes.
3. UHN Electronic Patient Records (EPR): Primary language, referral to CRS, and reason for CRS referral were extracted from the UHN EPR.

### 2.4 Sample Selection

A total of *n* = 2783 patients were referred to the CRS program from January 1, 2017 to December 31, 2019 and had clinical data available in the PM Cancer Registry database. To identify a comparator group of patients who were not referred to CRS, the cancer registry was then searched for all patients diagnosed with similar cancers (by primary site) during the same year and month. If an exact year/month match was not found, the search was expanded to include patients diagnosed within 3 months. In total, 18,626 un‐referred controls were identified. Of the 21,409 patients selected for analysis (referred and un‐referred), a single patient identified as transsexual was excluded as this did not allow for comparative purposes, and there were 2974 patients for whom postal code was unavailable, resulting in a final sample of 18,434. PROMs data were available for a sub‐group of 9499 patients, including 1585 patients referred to CRS. The most recent PROMs screening data prior to referral or, for un‐referred patients, the PROMs recorded nearest to the median of time from diagnosis to PROMs for the referred patients with the same cancer site was extracted.

### 2.5 Statistical Analysis

Summary statistics were used to describe the patients referred to the CRS program.

**FIGURE 1:**
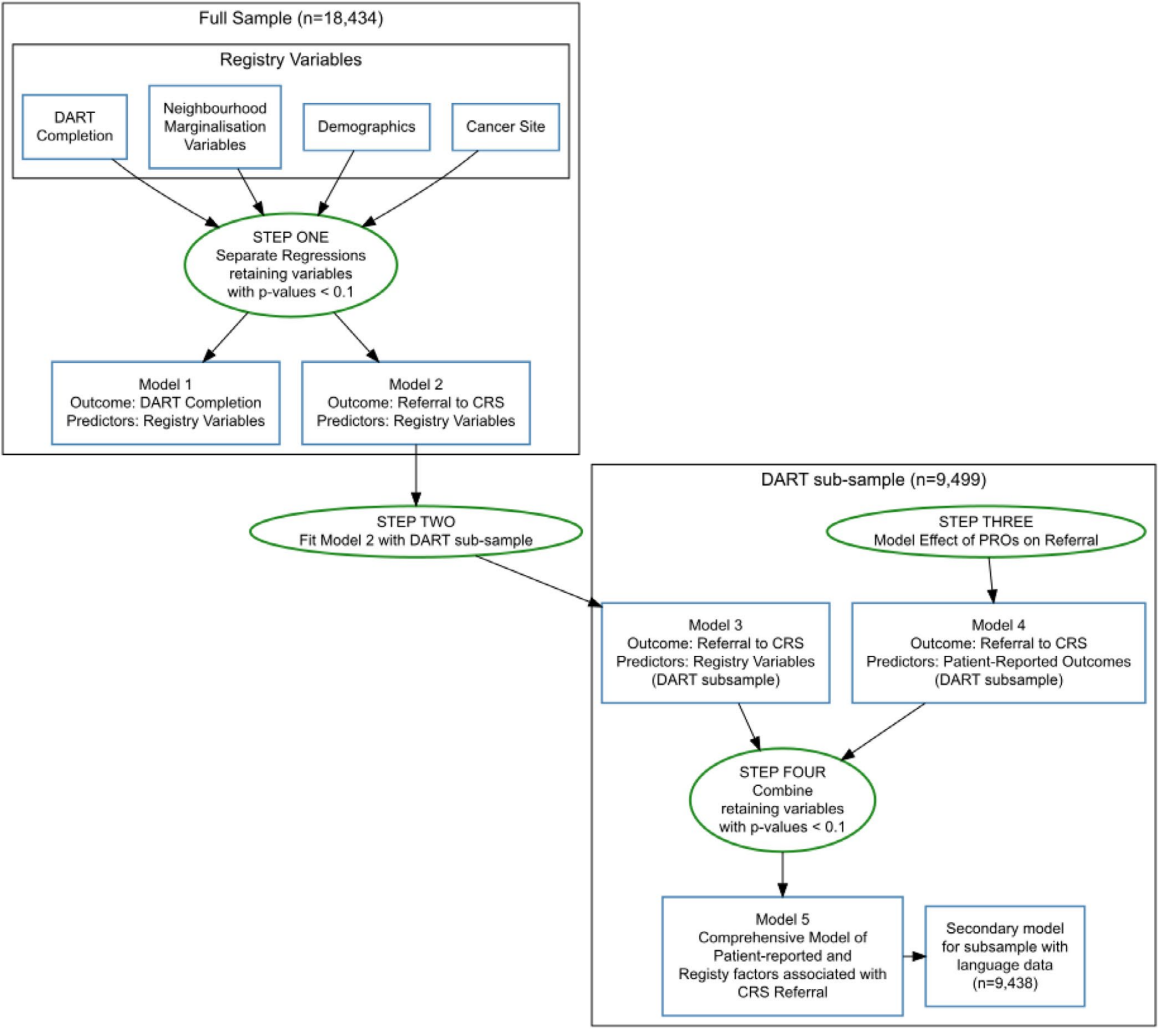
Model building process.

Our comparative analysis was conducted in several stages to allow us to identify important factors associated with CRS referral and to build a single comprehensive model incorporating sociodemographic and clinical characteristics and patient‐reported symptoms. This process is illustrated in Figure 1 and described here.

*Step one*: Because only half the sample completed the PROMS and there may be systematic differences between those who did and did not complete PROMs, we separately modeled the likelihood of completing a PROMS assessment (Model 1) and the likelihood of a referral to CRS (Model 2) using multivariable logistic regression modeling on all available sociodemographic and clinical data. Initially, separate analyses were conducted on groups of variables: demographic (age, sex, and distance from hospital), socio‐economic (income and marginalization variables) and cancer type (single variable); for Model 2, we also included a dichotomous indicator of whether the PROMs survey was completed.

*Step two*: We re‐fit Model 2 (likelihood of CRS referral) using the sub‐sample of patients who completed a PROMs assessment (Model 3) and compared the regression estimates.

*Step three*: For the sub‐sample of patients with PROMs data, we fit a model predicting CRS referral based on PROMs scores (Model 4). Model 3 was then augmented with the significant patient‐reported factors from Model 4 to create a comprehensive model of factors associated with CRS referral (Model 5). During the model building phase, factors were retained if the *p*‐value, unadjusted for multiple comparisons, was < 0.1. Acknowledging the exploratory nature of this work and the large sample size, a Holm's correction has been applied to the *p*‐values reported for each model.

*Step four*: To determine the relative importance of each variable in the comprehensive model, both the unique and univariate contributions of each predictor were estimated using the area under the curve (AUC). Unique AUC was assessed by subtracting the AUC of the model less the predictor from the full model AUC, and univariate AUC was calculated fitting a model with just the predictor. A variable importance plot was created by plotting each variable's unique contribution to the model AUC against the AUC of the variable in a univariate analysis.

Model assumptions were tested and verified using the DHARMa package [49] and all statistical analyses were conducted using the R statistical programming language version 4.2.3 [50].

## 3 Results

### 3.1 Characteristics of Patients Referred to CRS

**FIGURE 2:**
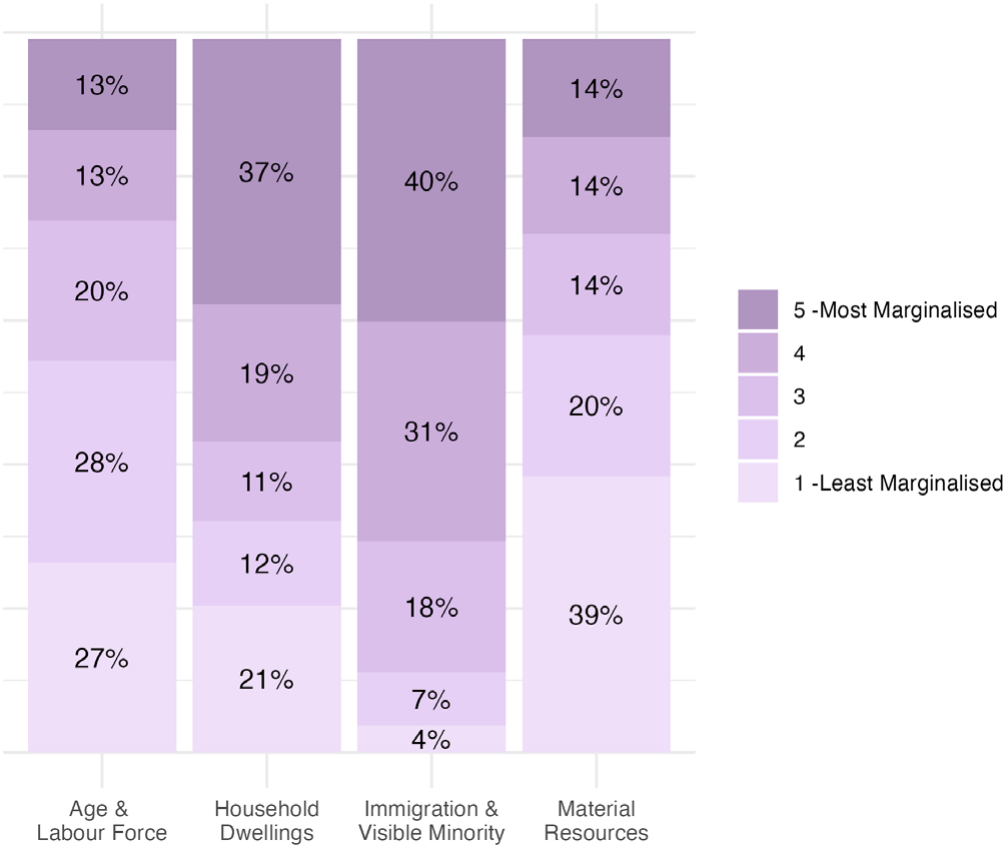
Distribution of marginalization characteristics for patients referred to the CRS program..

**FIGURE 3:**
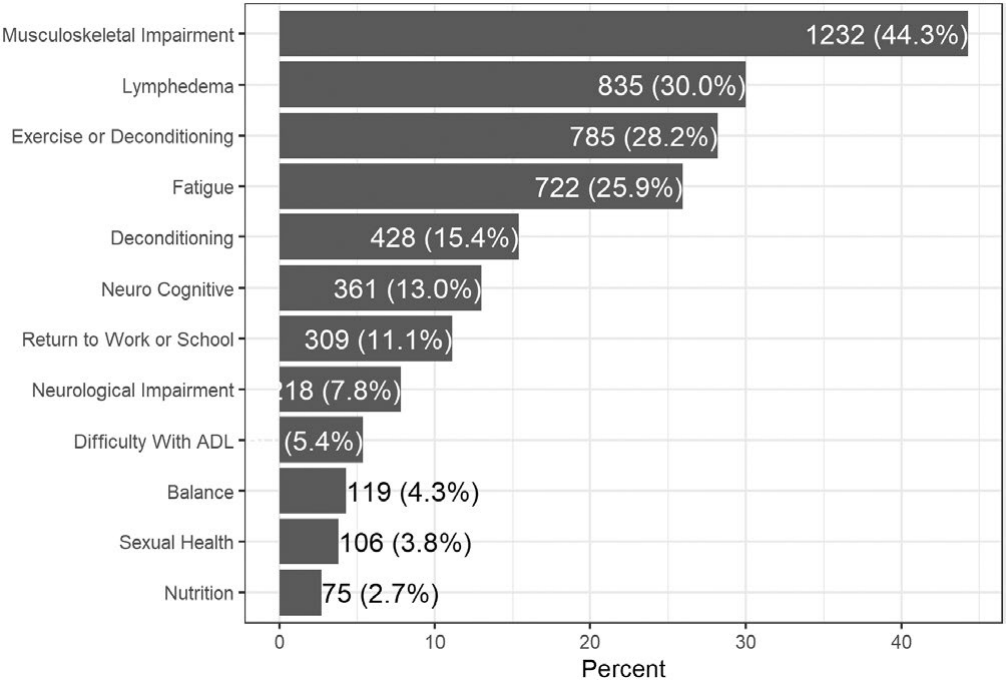
Most common reasons for referral to the CRS program.

Mean (standard deviation, SD) time from diagnosis to referral was 20.7 (19.2) months. The mean (SD) age of referred patients was 54.5 (13.8) years; 75% were female, 94% spoke English, and half (52%) lived within 15 km of the hospital. The most common site of cancer was breast (43.7%), followed by gynecological (12.9%) and lymphoma/myeloma (9.6%). Characteristics of the referred patients are presented in Table 1 and neighborhood attributes of referred patients are reported in Figure 2. Reasons for referral were recorded for *n* = 2702 (97%) of those referred. The most common reasons for referral were musculoskeletal impairment (44.3%) and lymphedema (30.0%). Referral reasons were not exclusive, and many patients (*n* = 1220, 45%) had multiple reasons for referral; Figure 3 provides a description of all reasons for referral. A PROMs assessment was available for 1807/2783 (65%) of people referred, of whom 1801 completed the ESAS. On the ESAS‐r, the most commonly reported moderate to severe symptoms [51] were: tiredness (47.3%) and lack of wellbeing (44.1%), followed by anxiety (32.4%), drowsiness (27.5%), pain (26.7%) and depression (26.5); fewer than 20% of patients reported moderate to severe lack of appetite, shortness of breath, or nausea. Symptom distributions for referred patients are reported in Table 2.

**TABLE 1:** Patient characteristics figures are *n* (%).

	All referred patients (*n* = 2783)	Matched referred patients (*n* = 2466)	Matched unreferred patients (*n* = 15,968)
Age at diagnosis			
Mean (SD)	54.5 (13.8)	54.5 (13.9)	60.7 (14.6)
Median (Q1, Q3)	54.8 (45.5, 64.2)	54.6 (45.5, 64.2)	62 (52, 71)
Sex			
Male	707 (25)	614 (25)	6173 (39)
Female	2076 (75)	1852 (75)	9795 (61)
English speaker			
No	180 [120]	165 (7)	844 (10)
Yes	2603 (94)	2301 (93)	7899 (90)
Distance from hospital (km)			
< 15	1423 (52)	1340 (54)	6790 (43)
15–49	1002 (37)	900 (36)	6495 (41)
50–99	181 (7)	146 (6)	1456 (9)
> 100	121 [121]	80 (3)	1227 (8)
Missing	56		
Living arrangements		706 (29)	3476 (22)
Lives with others	796 (29)	177 (7)	785 (5)
Lives alone	194 (7)	1583 (64)	11,707 (73)
Not reported	1793 (64)		
Patient ECOG (from PROMS)		349 (14)	3466 (22)
0	388 (14)	798 (32)	2849 (18)
1	889 (32)	215 (9)	655 (4)
2	252 [122]	131 (5)	491 (3)
3	155 [120]	10 (0)	60 (0)
4	10 (0)	963 (39)	8447 (53)
Not reported	1089 (39)		
Cancer site		1114 (45)	4848 (30)
Breast	1216 (43.7)	293 (12)	1894 (12)
Gynecological	358 (12.9)	239 (10)	2663 (17)
Lymphoma and myeloma	268 (9.6)	186 (8)	470 (3)
Head and neck	212 (7.6)	116 (5)	2594 (16)
Genitourinary	150 (5.4)	135 (5)	709 (4)
Gastrointestinal	138 (5)	83 (3)	255 (2)
Melanoma and skin	92 (3.3)	72 (3)	1011 (6)
Lung	80 (2.9)	49 (2)	167 (1)
Sarcoma	59 (2.1)	41 (2)	952 (6)
Endocrine	51 (1.8)	32 (1)	111 (1)
CNS and eye	36 (1.3)	15 (1)	38 (0)
Leukemia	21 (0.8)	91 (4)	256 (2)
Other	102 (3.7)	54.5 (13.9)	60.7 (14.6)
Treatments received			
Surgery	1714 (61.6)	1560 (63.3)	9408 (58.9)
Radiation	1697 (61.0)	1530 (62.0)	7022 (44.0)
Chemo	1644 (59.1)	1473 (59.7)	5039 (31.6)
Hormone	779 (28.0)	715 (29.0)	4120 (25.8)
Biological response modifiers (BRM)	371 (13.3)		1703 (10.7)

**TABLE 2:** ESAS symptom distributions for referred patients reported as *n* (%). Cases with missing values were removed from comparative models.

ESAS symptoms	None/Mild^a^	Moderate/Severe^b^	Missing
Tiredness	946 (52.5%)	851 (47.3%)	4 (0.2%)
Well‐being	1001 (55.6%)	794 (44.1%)	6 (0.3%)
Anxiety	1214 (67.4%)	583 (32.4%)	4 (0.2%)
Pain	1302 (72.3%)	496 (27.5%)	3 (0.2%)
Depression	1319 (73.2%)	481 (26.7%)	1 (0.1%)
Drowsiness	1323 (73.5%)	477 (26.5%)	1 (0.1%)
Lack of appetite	1487 (82.6%)	311 (17.3%)	3 (0.2%)
Shortness of breath	1531 (85.0%)	265 (14.7%)	5 (0.3%)
Nausea	1669 (92.7%)	129 (7.2%)	3 (0.2%)

^a^ Scores 0–3.

^b^ Scores 4–10.

### 3.2 Factors Affecting CRS Referral

The model building process is reported in Tables S1–S3 and Figures S2 and S3. Our comprehensive model predicting CRS referral (Model 5) from registry and patient reported factors is reported in Table 3. Older patients and those living furthest from the hospital were less likely to be referred, while those reporting pain or ECOG‐PRFS ≥ 1 were more likely to be referred. The likelihood of referral also varied by cancer site: relative to those with breast cancer, patients with head and neck cancer, melanoma, and skin and leukemia were more likely to be referred, and those with endocrine, genitourinary, gynecological, lymphoma, and myeloma and lung cancer were less likely to receive referrals.

**TABLE 3:** Multivariable model including registry and patient‐reported PROMS variables associated with referral to CRS. Holm's adjustment has been applied to *p*‐values to control for multiple comparisons (model 5).

	OR (95% CI)	*p*	*N*	Event
Age group (years)			9499	1585
40–64	Reference		5146	1002
< 40	1.93 (1.61, 2.32)	**< 0.001**	858	241
65+	0.48 (0.42, 0.55)	**< 0.001**	3495	342
Sex		0.12	9499	1585
Male	Reference		3568	399
Female	1.28 (1.05, 1.55)		5931	1186
Distance from hospital (km)			9499	1585
< 15	Reference		4224	854
15‐49	0.67 (0.59, 0.77)	**< 0.001**	3814	569
50–99	0.44 (0.35, 0.56)	**< 0.001**	839	103
> 100	0.38 (0.28, 0.51)	**< 0.001**	622	59
Age labor		0.34	9499	1585
Least engaged in labor force	Reference		4925	712
Most engaged in labor force	1.12 (0.99, 1.26)		4574	873
Cancer site			9499	1585
Breast	Reference		3009	694
CNS and eye	1.86 (0.88, 3.93)	0.35	36	12
Endocrine	0.23 (0.15, 0.36)	**< 0.001**	389	26
Gastrointestinal	1.34 (1.01, 1.79)	0.30	387	95
Genitourinary	0.32 (0.24, 0.44)	**< 0.001**	1633	77
Gynecological	0.72 (0.60, 0.86)	**0.003**	1346	221
Head and neck	2.24 (1.69, 2.99)	**< 0.001**	385	125
Leukemia	29.51 (6.16, 141.37)	**< 0.001**	13	11
Lung	0.52 (0.37, 0.72)	**0.001**	477	51
Lymphoma and myeloma	0.48 (0.38, 0.60)	**< 0.001**	1482	160
Melanoma and skin	2.55 (1.58, 4.11)	**0.001**	98	33
Sarcoma	1.49 (0.78, 2.82)	0.45	62	15
Other site	2.70 (1.88, 3.88)	**< 0.001**	182	65
Pain	1.06 (1.03, 1.09)	**< 0.001**	9499	1585
Anxiety	1.03 (1.00, 1.06)	0.35	9499	1585
Well‐being	1.04 (1.01, 1.08)	0.12	9499	1585
Shortness of breath	0.96 (0.93, 0.99)	0.11	9499	1585
ECOG			9499	1585
0	Reference		3773	346
1	2.30 (1.98, 2.67)	**< 0.001**	3619	796
2	2.46 (1.96, 3.08)	**< 0.001**	861	213
3	2.02 (1.54, 2.65)	**< 0.001**	613	130
4	1.23 (0.59, 2.56)	0.58	70	10
Not reported	1.31 (1.00, 1.72)	0.30	563	90

*Note:* Bold is highlighting statistically significant (0.05 findings).

**FIGURE 4:**
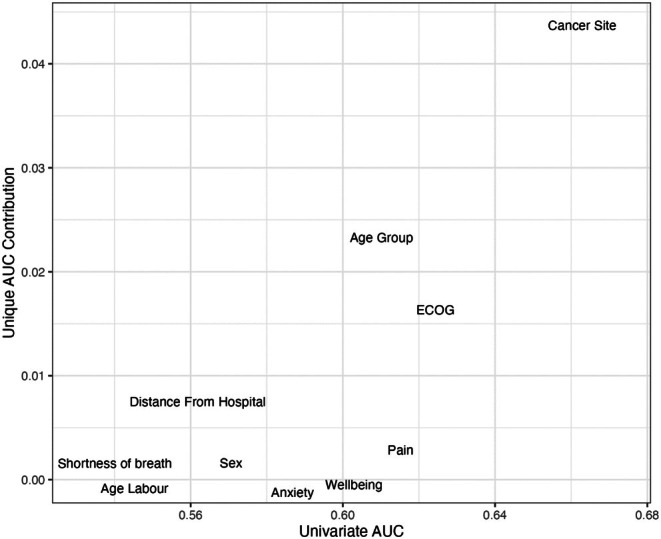
Variable importance plot of the registry and patient‐reported factors on the sub sample of patients who completed a PROMS assessment (model 5). Variables toward the right have the greatest predictive value in a univariate model, while those toward the top contribute most to the multivariable model.

A variable importance plot, detailing contributions for individual variables, as opposed to groups, for this combined model is shown in Figure 4 and indicates that cancer site, patient‐reported performance status (ECOG‐PRFS) and age are the strongest unique predictors of referral to CRS. Contributions of the variable blocks are described in Table 4. A sensitivity analysis including patients with available language information is reported in Table S4; results are similar to the main findings. Although patients who spoke English were, on average, more likely to be referred, the effect was not statisticallysignificant.

**TABLE 4:** Model contribution statistics for variable blocks from the complete sample of patients in the hospital cancer registry (model 2, *n* = 18,434). Variance explained is calculated as Nagelkerke's pseudo‐*R* ^2^ and the contribution to the full model is the full model AUC less the AUC of the full model fit without the block.

Variable block	AUC	Variance explained	Contribution to full model
Full registry model	0.74	0.15	
Demographics	0.66	0.07	0.05
Canadian Marginalization Index	0.57	0.01	0.00
Cancer type	0.67	0.08	0.07
PROMS completed	0.57	0.02	0.02

Because PROMS completion is itself a predictor of CRS referral it is important to understand which groups of patients are more likely to complete a PROM. Table S1 indicates that older patients are less likely to complete a PROM while patients from neighborhoods with the most material resources and those with incomes in the top 20% are more likely to complete the PROMS. There is also variability across cancer sites, relative to those with breast cancer (the largest group) those with gynecological, genitourinary and head and neck cancers are more likely to complete the PROMS, while CNS and eye, endocrine, leukemia and melanoma and sarcoma patients are less likely to do so.

## 4 Discussion

Cancer rehabilitation is an essential part of cancer care, but it is often underutilized. We undertook a large retrospective analysis at Canada's largest cancer center in order to describe the characteristics of people referred to cancer rehabilitation and to explore associations between demographic and clinical variables and PROMs with referral to CRS.

We found that the average age of those referred was 55 years, which is lower than the average age of those diagnosed with cancer in Canada (67 years) and lower than those at PM who were not referred (60.4 years) [52]. The majority of people referred to CRS were female (75%) and spoke English (95%). The most frequently referred cancer site was breast, followed by gynecological and lymphoma/myeloma, and the most common reasons for referral were musculoskeletal impairment and lymphedema. Referral reasons were not exclusive, and many patients (45%) had multiple reasons for referral, highlighting the need for holistic comprehensive assessment and multidimensional rehabilitation [26, 53].

Several key predictors of CRS referral were identified, including cancer site, lower age (< 65 years), closer distance to hospital, poorer patient‐reported functional status, and higher pain scores. These findings indicate the need for targeted processes to increase accessibility to cancer rehabilitation, particularly for older patients and those living further from the hospital. In contrast to previous research in the USA [39], neighborhood‐level socioeconomic data did not contribute uniquely to the referral model. The differences in findings may be due to socialized medical systems versus insurer‐based systems where cost can be a barrier to access [54]. Additionally, we found that completing PROMs increased the likelihood of CRS referral, but that PROMs completion rates also varied by demographic and cancer site factors. Older patients were less likely to complete the PROMs, while those from neighborhoods with higher material resources and incomes were more likely. Cancer type also influenced PROMs completion. Given that the odds of CRS referral based on demographic and clinical factors did not change when we restricted our sample to those who completed a PROMs, it is important to consider and address both the factors associated with CRS referral among PROMs responders and factors associated with PROMs response to fully understand which groups of people are being referred and who may be excluded. Interestingly, a recent systematic review and meta‐analysis of cardiac, pulmonary, and ICU rehabilitation/recovery programs found very similar barriers to referral, including transportation, distance, work conflicts, and patient factors (e.g., comorbidities, older age) while an important institutional facilitator to referral included provider recommendations for program participation [55].

Differences in referral rates across disease sites may be due to a number of factors. To begin, impairments can differ across disease sites, with some more amenable to outpatient cancer rehabilitation [27, 56, 57, 58, 59, 60]. There may also be differences related to the culture within the clinics. For example, some clinics have larger volumes, and cancer rehabilitation may not be deemed a priority during time‐constrained office visits [61, 62, 63]. Referral rates to cancer rehabilitation can also be influenced by oncologists' personal knowledge and attitudes. While oncologists generally agree that cancer rehabilitation is necessary [64, 65], they often under‐detect symptoms or underestimate their severity and have difficulty identifying physical dysfunction [64, 66, 67, 68, 69]. Further, oncologists may be unaware of the benefits of cancer rehabilitation [70, 71, 72, 73] and unfamiliar with programs and how to refer [74]. In a recent ecological review of implementation barriers to exercise in oncology, HCPs reported uncertainty about the safety and quality of exercise as a barrier and described hesitation referring patients to exercise who were previously inactive, elderly, or undergoing treatment [75]. This can result in inequities in access because patients rely on oncologists for referrals to cancer rehabilitation programs [26]. In fact, the strength of the HCPs recommendation for supportive care, including rehabilitation, is one of the most important factors affecting uptake [76, 77]. To enhance oncologists' knowledge about and referrals to cancer rehabilitation, residency and fellowship programs can include more within the curriculum, including shadowing experiences with physiatrists and cancer rehabilitation programs, helping future oncologists better understand the functional challenges cancer patients face and the benefits of cancer rehabilitation [64, 69]. Additionally, including cancer rehabilitation specialists (i.e., physiatry, physiotherapy, occupational therapy) in site‐specific tumor boards and case rounds may help to promote the consideration of rehabilitation and ensure that the functional impacts of cancer diagnoses and treatments are addressed early [64, 78].

Older age is associated with both functional decline and also cancer risk [79, 80, 81]. Up to half of older adults with cancer require assistance with one or more activities of daily living (ADLs), and 75% need help with instrumental activities of daily living (IADLs), such as managing medications or preparing meals [82]. Difficulties with ADLs and IADLs can limit their ability to engage in social, leisure, and work activities, affecting their roles and overall quality of life [83, 84, 85]. Further, impaired physical function has been shown to increase the risk of hospitalization and long‐term care in older adults with cancer [86]. Despite this, we found that older adults were less likely to be referred to cancer rehabilitation. A lower referral rate in older adults has been reported by others along with findings that older adults who express rehabilitation needs are less likely to have them addressed [87, 88, 89]. The barriers to access for this population are likely multifactorial, including both health care provider (HCP) and patient factors. For example, studies have reported that physicians may have concerns about the safety of rehabilitation given the perceived risks of complications in this age group and may be hesitant to refer older patients with multiple health conditions or shorter life expectancies, assuming they may not benefit as much or be able to actively participate [90]. However, research has demonstrated that rehabilitation and exercise interventions are safe and feasible [91] and can reduce disability in older patients [92] including those with cancer [93, 94, 95, 96, 97]. In addition to potential HCP factors, many older adults face physiological barriers such as symptom burden and other comorbidities that may limit their ability to engage in rehabilitation programs [98, 99], barriers such as low self‐efficacy and motivation [100], and practical limitations such as the inability to drive or arrange transportation, making access difficult [100].

Patients who lived further away from the cancer center were also less likely to be referred to cancer rehabilitation. It is possible that oncologists did not refer patients that they knew were coming from far distances given the challenge to overcome the time, energy, and costs required to travel for those who are already dealing with cancer‐related impairments [101]. The use of technology to deliver cancer rehabilitation (tele‐rehabilitation) can be one effective way to reduce some of these barriers and has been shown to be feasible and acceptable [102] and effective in reducing disability [103] and improving quality of life [104]. Further, tele‐rehabilitation has been shown to be effective and acceptable in older adult populations [105, 106, 107]. In fact, the PM CRS program has developed a home‐based cancer rehabilitation program called CaRE@Home [42] which aims to increase access to cancer rehabilitation for those who experience barriers to hospital‐based services, and since COVID‐19, we now offer many of our consults virtually [108].

Patient reported function status and greater pain scores were both associated with CRS referral. Interestingly, the completion of the PROMs measures also significantly increased one's odds of being referred. This finding adds to the growing body of evidence that the integration of PROMs into cancer care can help in the detection of symptoms and side effects resulting in improved clinical management [109, 110]. PROMs are used to capture patients' views on their health, along with their needs, preferences, and values [111]. Numerous studies have shown that incorporating PROMs in standard clinical practice enhances communication between patients and providers, boosts patient satisfaction, and may improve the monitoring of treatment responses and the identification of previously unrecognized issues [112].

Strengths of the study include a large sample obtained from Canada's largest cancer center which offers robust supportive care services including cancer rehabilitation. The sample size allowed for multiple comparisons across sociodemographic and clinical factors. The inclusion of PROMs data, which is routinely collected at PM, is unique and allowed for exploration of the unique contribution of the individual scores but also whether the use of PROMs can drive referrals. Despite these strengths, these results need to be considered in relation to the limitations of the study design. This was a retrospective study and limited to the variables available though the PM registry, PROMs database, and EPR. Further, PM is a single comprehensive cancer centre located in Canada's largest city, which may limit generalizability. The study period was also 2017–2019, chosen purposefully to avoid the impact of COVID‐19, and the referral patterns may have shifted since this time. Finally, while our study explored predictors of CRS referral, our approach did not capture the specific barriers in the pathway to referral, including CRS eligibility, offer of referral by a physician, and patient acceptance. Future in‐depth research examining barriers and facilitators to referral along this pathway will be helpful in developing targeted approaches.

## 5 Conclusion

Despite the high documented rates of cancer‐related impairments and disability, referrals to cancer rehabilitation services have been reportedly low. In the current study, we found that, in addition to function, the most important predictors of referral were cancer type, age, and distance from the hospital. In addition, the completion of in‐clinic screening measures (PROMs) was also related to a higher likelihood of being referred. While the findings from this study warrant further exploration, including a more in‐depth examination of barriers, patient preferences, HCP attitudes, and other factors that may contribute to differences in referral, insights from this study can be helpful in optimizing the referral processes and addressing disparities regarding access to cancer rehabilitation services. Solutions are likely multifaceted, including HCP and patient education, as well as systemic changes to address barriers to referral. These may include increasing awareness of the importance of cancer rehabilitation services, the development and implementation of tele‐rehabilitation services, and addressing barriers to access among older adults with cancer. Affecting the culture around completing PROMs screening tools across the different cancer sites and with older patients will also likely have an important impact on referral rates.

## Author Contributions

**Jennifer M. Jones:** conceptualization (lead), data curation (lead), methodology (lead), project administration (lead), resources (lead), supervision (lead), writing – original draft (equal), writing – review and editing (equal). **Rogih Andrawes:** conceptualization (equal), data curation (equal), investigation (equal), methodology (equal), writing – original draft (equal), writing – review and editing (equal). **Michelle A. Weller:** conceptualization (equal), data curation (equal), investigation (equal), methodology (equal), project administration (equal), writing – original draft (equal), writing – review and editing (equal). **Adrienne Lam:** data curation (supporting), investigation (supporting), methodology (supporting), writing – original draft (supporting), writing – review and editing (equal). **Gilla K. Shapiro:** conceptualization (supporting), data curation (supporting), investigation (equal), methodology (equal), writing – review and editing (equal). **Madeline Li:** conceptualization (supporting), investigation (supporting), methodology (supporting), resources (supporting), writing – review and editing (equal). **Danielle Rodin:** conceptualization (supporting), investigation (supporting), methodology (supporting), writing – review and editing (equal). **Lisa Avery:** conceptualization (equal), data curation (equal), formal analysis (lead), investigation (equal), methodology (equal), supervision (supporting), writing – original draft (equal), writing – review and editing (equal).

## Disclosure

The authors have nothing to report.

## Conflicts of Interest

The authors declare no conflicts of interest.

## Supporting information


Data S1.

## Data Availability

Data is available on request and following approval from UHN Research Ethics Board.
