# Supplementary material for: A Case Control Study Examining the Patterns and Predictors of Referral to Cancer Rehabilitation at Canada's Largest Comprehensive Cancer Centre
Source: Cancer Med. 2025 Jul 10;14(13):e71046. doi: 10.1002/cam4.71046 (PMC12242712; doi:10.1002/cam4.71046)
Supplement: Supplementary file 1 — Data S1. [file CAM4-14-e71046-s001.docx]

Supplemental Material

Table of Contents

[Supplemental Section 1– SES Variable Categorisation 2](#_Toc185415061)

[Socioeconomic Variables Preliminary Model 2](#_Toc185415062)

[Supplemental Section 2– Model Building 3](#_Toc185415063)

[Supplemental Section 3: Sensitivity Model including Patient Language 8](#_Toc185415064)

[Supplemental Section 4: On-Marg domain proportions & Referral 9](#_Toc185415065)

# Supplemental Section 1– SES Variable Categorisation

### Socioeconomic Variables Preliminary Model

Based on the odds ratios in the forest plot from a multivariable model with all socioeconomic variables suggests the following dichotomisations (to create positive odds ratios):

| Domain | Reference Group(s) | Comparison Group(s) |
| --- | --- | --- |
| Age & Labour Force | Quintiles 3-5 | Quintiles 1-2 |
| Material Resources | Quintiles 3-5 | Quintiles 1-2 |
| Household Dwellings | Quintiles 3-5 | Quintiles 1-2 |
| Immigration & Visible Minority | Quintiles 1-2 | Quintiles 3-5 |
| CMA Income | Quintiles 1-4 | Quintile 5 |
| 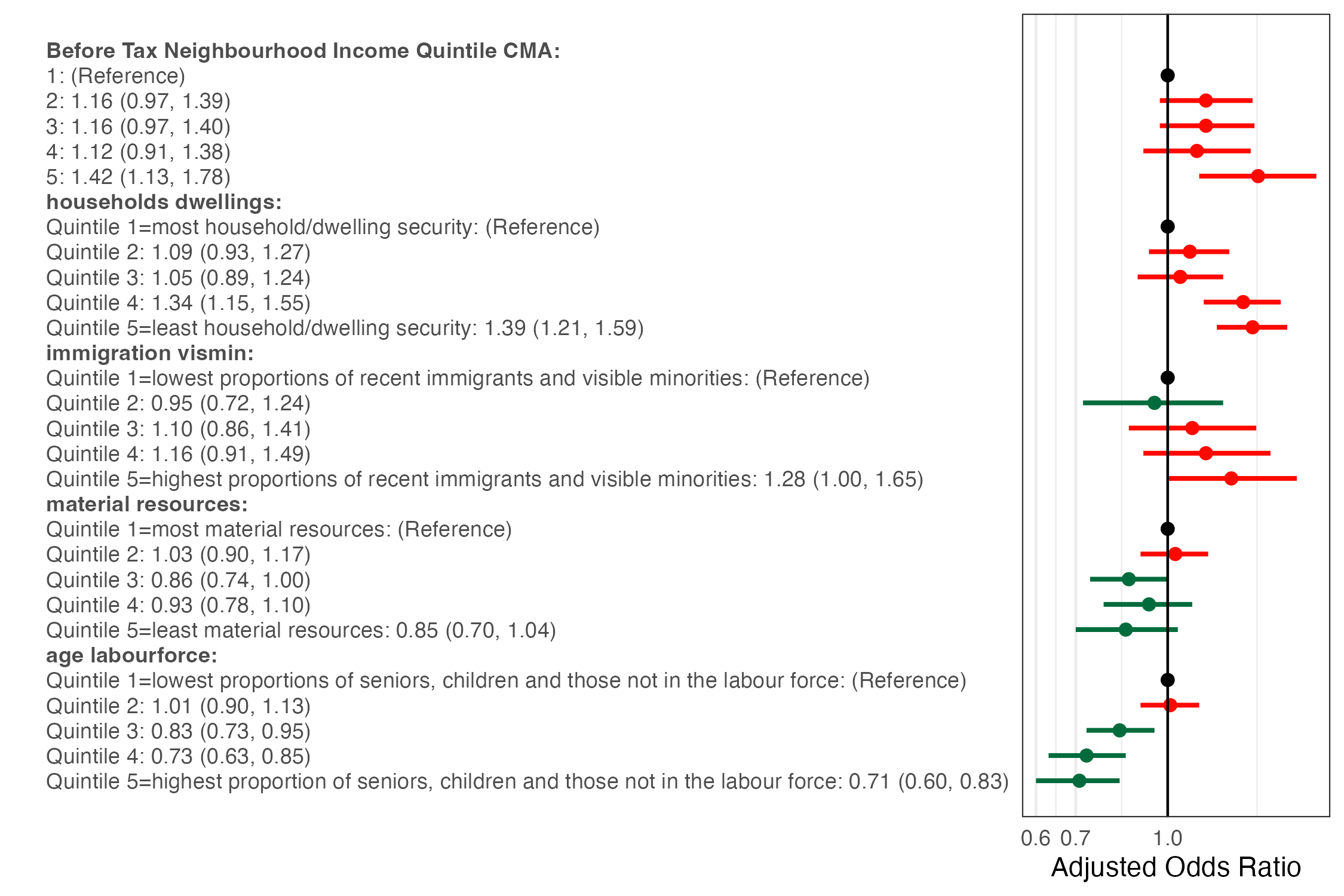  Figure S1: Odds ratios of CRS referral vs non-referral for the complete sample (n=19,491) for a multivariable model of Ontario marginalization indices. | | |

# Supplemental Section 2– Model Building

Table S1 Multivariable model of registry-available variables on likelihood of completing DART screening (Model 1).

|  | OR(95%CI) | p-value | N | Event |
| --- | --- | --- | --- | --- |
| **Age Group** |  |  | 18434 | 9672 |
| 40-64yrs | Reference |  | 9279 | 5221 |
| <40yrs | 0.96 (0.86, 1.07) | 1.00 | 1669 | 874 |
| 65yrs+ | 0.67 (0.63, 0.71) | **<0.001** | 7486 | 3577 |
| **Sex** |  | 1.00 | 18434 | 9672 |
| Male | Reference |  | 6787 | 3631 |
| Female | 0.94 (0.86, 1.04) |  | 11647 | 6041 |
| **Distance From Hospital** |  |  | 18434 | 9672 |
| <15km | Reference |  | 8130 | 4303 |
| 15-49km | 1.06 (0.98, 1.13) | 1.00 | 7395 | 3884 |
| 50-99km | 1.03 (0.91, 1.15) | 1.00 | 1602 | 851 |
| >100km | 0.89 (0.78, 1.02) | 0.81 | 1307 | 634 |
| **Material Resources** |  | **<0.001** | 18434 | 9672 |
| Least material resources | Reference |  | 8463 | 4089 |
| Most material resources | 1.28 (1.19, 1.38) |  | 9971 | 5583 |
| **Age Labour** |  | 1.00 | 18434 | 9672 |
| Least engaged in labour force | Reference |  | 9671 | 5025 |
| Most engaged in labour force | 0.96 (0.91, 1.03) |  | 8763 | 4647 |
| **Immigration Visible Minority** |  | 1.00 | 18434 | 9672 |
| Lowest proportion of immigrants | Reference |  | 2481 | 1273 |
| Highest proportion of immigrants | 1.06 (0.96, 1.17) |  | 15953 | 8399 |
| **Income** |  | **0.004** | 18434 | 9672 |
| Bottom 80% of income in CMA | Reference |  | 11935 | 6009 |
| Top 20% of income in CMA | 1.16 (1.07, 1.26) |  | 6499 | 3663 |
| **Cancer Site** |  |  | 18434 | 9672 |
| Breast | Reference |  | 5962 | 3059 |
| CNS and Eye | 0.31 (0.21, 0.46) | **<0.001** | 143 | 36 |
| Endocrine | 0.60 (0.52, 0.69) | **<0.001** | 993 | 396 |
| Gastrointestinal | 0.88 (0.75, 1.03) | 0.87 | 844 | 393 |
| Genitourinary | 1.54 (1.35, 1.75) | **<0.001** | 2710 | 1650 |
| Gynaecological | 1.70 (1.53, 1.88) | **<0.001** | 2187 | 1371 |
| Head and Neck | 1.43 (1.20, 1.71) | **0.001** | 656 | 389 |
| Leukaemia | 0.31 (0.16, 0.58) | **0.004** | 53 | 13 |
| Lung | 0.88 (0.76, 1.01) | 0.79 | 1083 | 487 |
| Lymphoma and Myeloma | 1.10 (0.99, 1.22) | 0.81 | 2902 | 1528 |
| Melanoma and Skin | 0.41 (0.32, 0.52) | **<0.001** | 338 | 99 |
| Sarcoma | 0.40 (0.30, 0.54) | **<0.001** | 216 | 64 |
| Other Site | 1.18 (0.94, 1.48) | 1.00 | 347 | 187 |


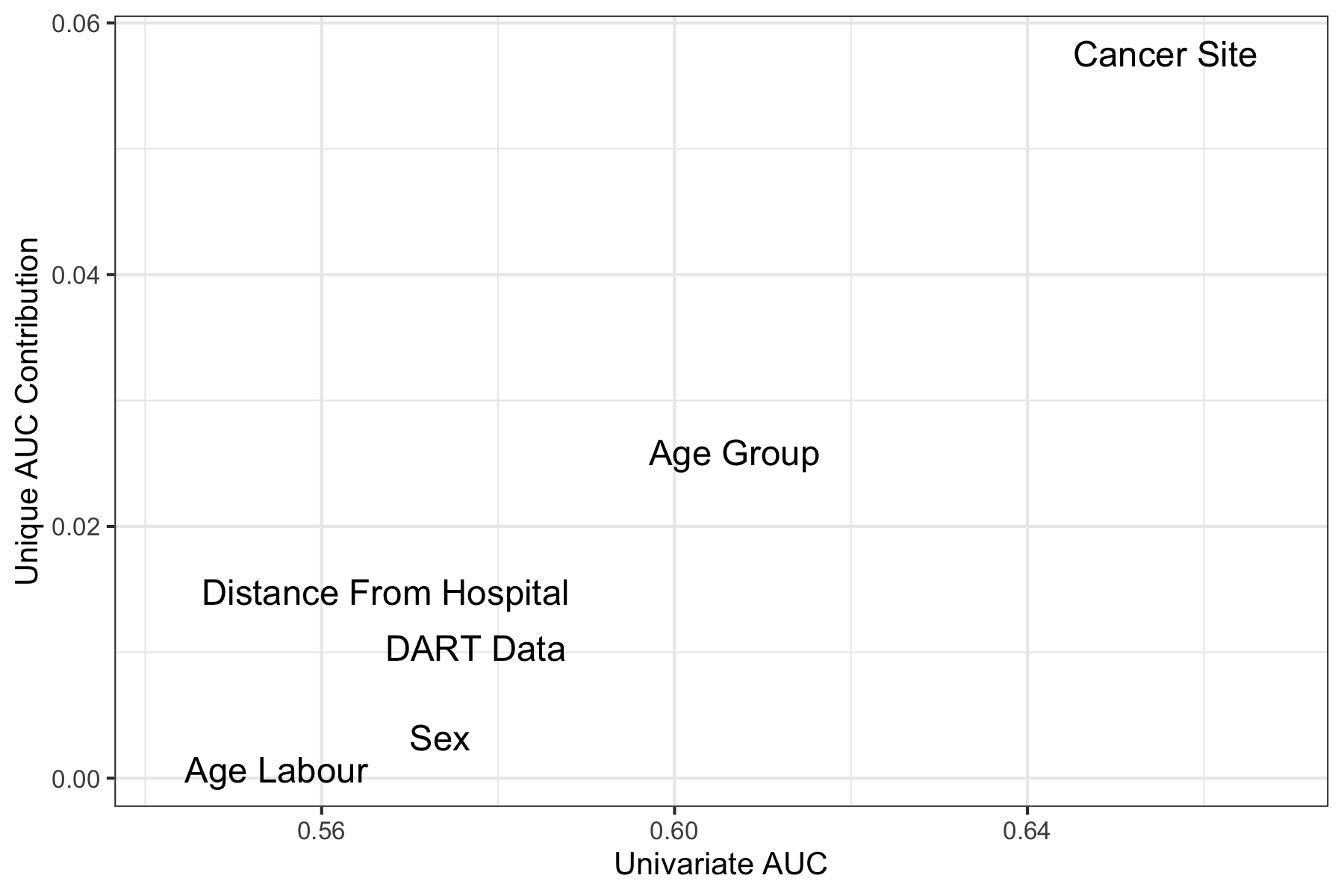


Figure S2: Variable importance plot for the Model 2, a model predicting CRS referral from the registry-reported factors for the entire sample.

Table S2 Multivariable model of registry-collected factors associated with referral to CRS. Holm’s adjustment has been applied to p-values to control for multiple comparisons (Model 2).

|  | OR(95%CI) | p-value | N | Event |
| --- | --- | --- | --- | --- |
| **Age Group** |  |  | 18434 | 2466 |
| 40-64yrs | Reference |  | 9279 | 1514 |
| <40yrs | 1.76 (1.53, 2.02) | **<0.001** | 1669 | 375 |
| 65yrs+ | 0.46 (0.42, 0.52) | **<0.001** | 7486 | 577 |
| **Sex** |  | **<0.001** | 18434 | 2466 |
| Male | Reference |  | 6787 | 614 |
| Female | 1.37 (1.19, 1.59) |  | 11647 | 1852 |
| **Distance From Hospital** |  |  | 18434 | 2466 |
| <15km | Reference |  | 8130 | 1340 |
| 15-49km | 0.69 (0.62, 0.76) | **<0.001** | 7395 | 900 |
| 50-99km | 0.42 (0.34, 0.50) | **<0.001** | 1602 | 146 |
| >100km | 0.30 (0.24, 0.39) | **<0.001** | 1307 | 80 |
| **Age Labour** |  | **0.015** | 18434 | 2466 |
| Least engaged in labour force | Reference |  | 9671 | 1111 |
| Most engaged in labour force | 1.13 (1.03, 1.24) |  | 8763 | 1355 |
| **Cancer Site** |  |  | 18434 | 2466 |
| Breast | Reference |  | 5962 | 1114 |
| CNS and Eye | 1.97 (1.29, 3.02) | **0.005** | 143 | 32 |
| Endocrine | 0.19 (0.14, 0.26) | **<0.001** | 993 | 41 |
| Gastrointestinal | 1.33 (1.06, 1.65) | **0.015** | 844 | 135 |
| Genitourinary | 0.31 (0.25, 0.40) | **<0.001** | 2710 | 116 |
| Gynaecological | 0.70 (0.61, 0.81) | **<0.001** | 2187 | 293 |
| Head and Neck | 2.91 (2.34, 3.62) | **<0.001** | 656 | 186 |
| Leukaemia | 3.56 (1.90, 6.68) | **<0.001** | 53 | 15 |
| Lung | 0.51 (0.39, 0.66) | **<0.001** | 1083 | 72 |
| Lymphoma and Myeloma | 0.53 (0.45, 0.63) | **<0.001** | 2902 | 239 |
| Melanoma and Skin | 2.58 (1.95, 3.41) | **<0.001** | 338 | 83 |
| Sarcoma | 1.94 (1.36, 2.75) | **<0.001** | 216 | 49 |
| Other Site | 2.28 (1.73, 3.00) | **<0.001** | 347 | 91 |
| **DART Data** |  | **<0.001** | 18434 | 2466 |
| No DART | Reference |  | 8762 | 861 |
| DART Completed | 1.88 (1.72, 2.07) |  | 9672 | 1605 |

*Table S3 Multivariable model of registry available factors associated with CRS referral on the sub-sample of patients for whom a DART was completed (n=10,307, Model 3)*

|  | OR(95%CI) | p-value | N | Event |
| --- | --- | --- | --- | --- |
| **Age Group** |  |  | 9499 | 1585 |
| 40-64yrs | Reference |  | 5146 | 1002 |
| <40yrs | 1.80 (1.51, 2.15) | **<0.001** | 858 | 241 |
| 65yrs+ | 0.47 (0.41, 0.53) | **<0.001** | 3495 | 342 |
| **Sex** |  | **0.005** | 9499 | 1585 |
| Male | Reference |  | 3568 | 399 |
| Female | 1.37 (1.14, 1.66) |  | 5931 | 1186 |
| **Distance From Hospital** |  |  | 9499 | 1585 |
| <15km | Reference |  | 4224 | 854 |
| 15-49km | 0.69 (0.61, 0.78) | **<0.001** | 3814 | 569 |
| 50-99km | 0.45 (0.36, 0.57) | **<0.001** | 839 | 103 |
| >100km | 0.39 (0.29, 0.53) | **<0.001** | 622 | 59 |
| **Age Labour** |  | 0.094 | 9499 | 1585 |
| Least engaged in labour force | Reference |  | 4925 | 712 |
| Most engaged in labour force | 1.14 (1.01, 1.28) |  | 4574 | 873 |
| **Cancer Site** |  |  | 9499 | 1585 |
| Breast | Reference |  | 3009 | 694 |
| CNS and Eye | 2.16 (1.05, 4.46) | 0.094 | 36 | 12 |
| Endocrine | 0.23 (0.15, 0.35) | **<0.001** | 389 | 26 |
| Gastrointestinal | 1.63 (1.24, 2.16) | **0.003** | 387 | 95 |
| Genitourinary | 0.29 (0.22, 0.40) | **<0.001** | 1633 | 77 |
| Gynaecological | 0.73 (0.62, 0.87) | **0.003** | 1346 | 221 |
| Head and Neck | 2.75 (2.08, 3.64) | **<0.001** | 385 | 125 |
| Leukaemia | 32.48 (7.08, 149.11) | **<0.001** | 13 | 11 |
| Lung | 0.63 (0.46, 0.87) | **0.019** | 477 | 51 |
| Lymphoma and Myeloma | 0.55 (0.44, 0.68) | **<0.001** | 1482 | 160 |
| Melanoma and Skin | 2.76 (1.75, 4.37) | **<0.001** | 98 | 33 |
| Sarcoma | 1.49 (0.80, 2.77) | 0.21 | 62 | 15 |
| Other Site | 3.01 (2.12, 4.29) | **<0.001** | 182 | 65 |

The odds of referral are generally similar for those who did and did not complete the DART. The main exception is leukemia patients, who are much more likely to be referred with a DART - however, leukemia patients are also less likely to complete a DART, only 13 patients with leukemia completed a DART, and of these 11 were referred.


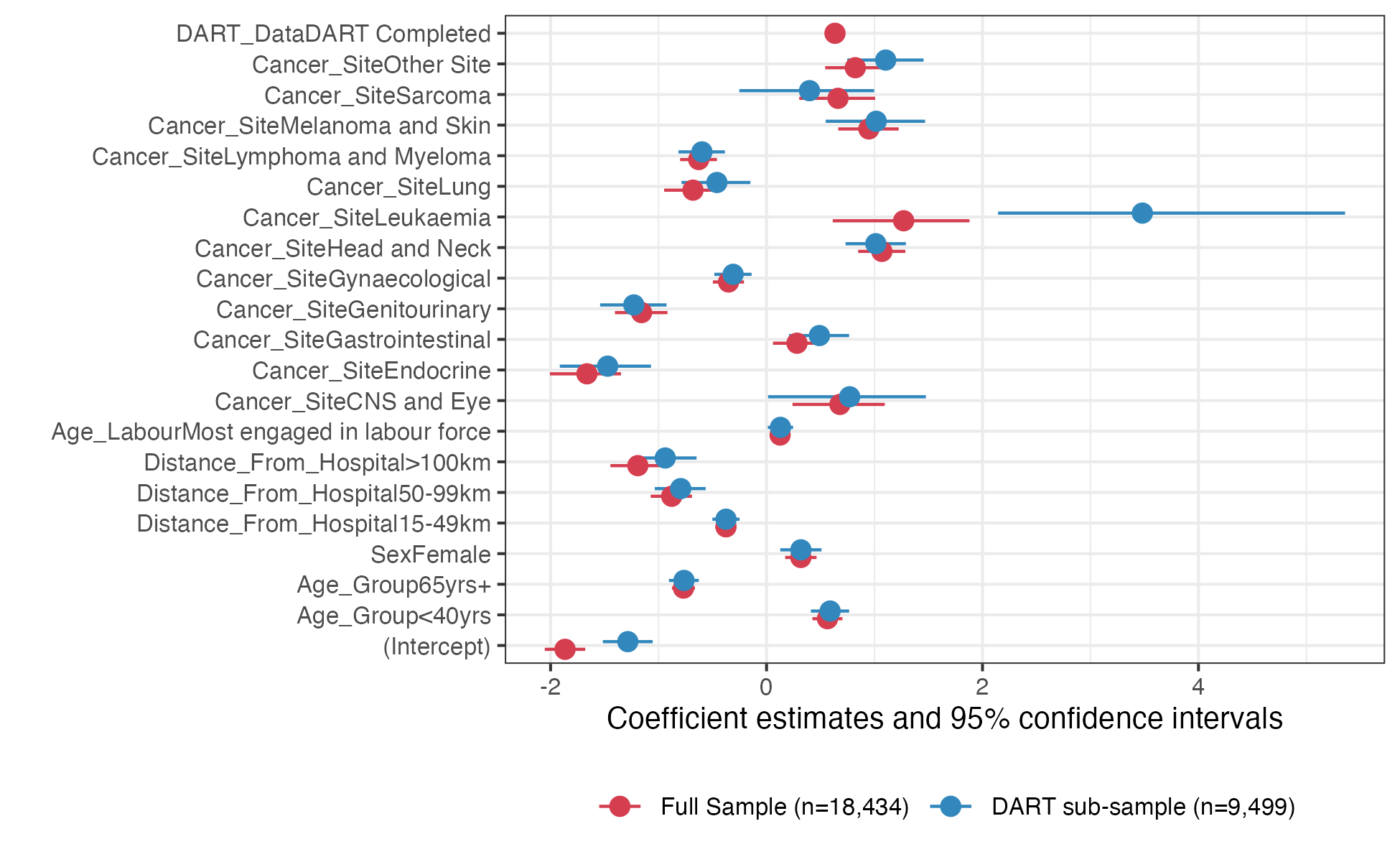


Figure S3: Comparison of the raw (un-transformed) estimates from Models 2 and 3. Patients with Leukemia are more likely to be referred if a DART

# Supplemental Section 3: Sensitivity Model including Patient Language

Table S4 A sensitivity model including as an additional predictor whether the patient spoke English. This model was fit removing the 61 patients without language data.

|  | OR(95%CI) | p-value | N | Event |
| --- | --- | --- | --- | --- |
| **Age Group** |  |  | 9438 | 1585 |
| 40-64yrs | Reference |  | 5115 | 1002 |
| <40yrs | 1.92 (1.60, 2.30) | **<0.001** | 852 | 241 |
| 65yrs+ | 0.49 (0.43, 0.57) | **<0.001** | 3471 | 342 |
| **Sex** |  | 0.12 | 9438 | 1585 |
| Male | Reference |  | 3534 | 399 |
| Female | 1.28 (1.05, 1.55) |  | 5904 | 1186 |
| **Distance From Hospital** |  |  | 9438 | 1585 |
| <15km | Reference |  | 4207 | 854 |
| 15-49km | 0.68 (0.60, 0.77) | **<0.001** | 3792 | 569 |
| 50-99km | 0.44 (0.35, 0.56) | **<0.001** | 826 | 103 |
| >100km | 0.37 (0.28, 0.51) | **<0.001** | 613 | 59 |
| **Age Labour** |  | 0.32 | 9438 | 1585 |
| Least engaged in labour force | Reference |  | 4888 | 712 |
| Most engaged in labour force | 1.12 (0.99, 1.26) |  | 4550 | 873 |
| **Cancer Site** |  |  | 9438 | 1585 |
| Breast | Reference |  | 3009 | 694 |
| CNS and Eye | 1.92 (0.91, 4.06) | 0.32 | 36 | 12 |
| Endocrine | 0.23 (0.15, 0.36) | **<0.001** | 389 | 26 |
| Gastrointestinal | 1.35 (1.01, 1.80) | 0.28 | 387 | 95 |
| Genitourinary | 0.32 (0.24, 0.44) | **<0.001** | 1633 | 77 |
| Gynaecological | 0.73 (0.61, 0.87) | **0.005** | 1346 | 221 |
| Head and Neck | 2.24 (1.68, 2.98) | **<0.001** | 385 | 125 |
| Leukaemia | 28.64 (5.98, 137.21) | **<0.001** | 13 | 11 |
| Lung | 0.52 (0.38, 0.73) | **0.002** | 476 | 51 |
| Lymphoma and Myeloma | 0.50 (0.40, 0.62) | **<0.001** | 1426 | 160 |
| Melanoma and Skin | 2.53 (1.57, 4.07) | **0.002** | 98 | 33 |
| Sarcoma | 1.48 (0.78, 2.81) | 0.46 | 62 | 15 |
| Other Site | 2.78 (1.93, 4.01) | **<0.001** | 178 | 65 |
| **Pain** | 1.06 (1.03, 1.09) | **<0.001** | 9438 | 1585 |
| **Anxiety** | 1.03 (1.00, 1.06) | 0.32 | 9438 | 1585 |
| **Wellbeing** | 1.04 (1.01, 1.08) | 0.12 | 9438 | 1585 |
| **Shortness of breath** | 0.96 (0.93, 0.99) | 0.12 | 9438 | 1585 |
| **ECOG** |  |  | 9438 | 1585 |
| 0 | Reference |  | 3759 | 346 |
| 1 | 2.30 (1.98, 2.67) | **<0.001** | 3595 | 796 |
| 2 | 2.45 (1.95, 3.07) | **<0.001** | 854 | 213 |
| 3 | 2.04 (1.55, 2.68) | **<0.001** | 605 | 130 |
| 4 | 1.25 (0.60, 2.62) | 0.55 | 68 | 10 |
| Not Reported | 1.33 (1.01, 1.74) | 0.28 | 557 | 90 |
| **English Speaker** |  | 0.081 | 9438 | 1585 |
| No | Reference |  | 847 | 97 |
| Yes | 1.38 (1.09, 1.74) |  | 8591 | 1488 |

# Supplemental Section 4: On-Marg domain proportions & Referral

*
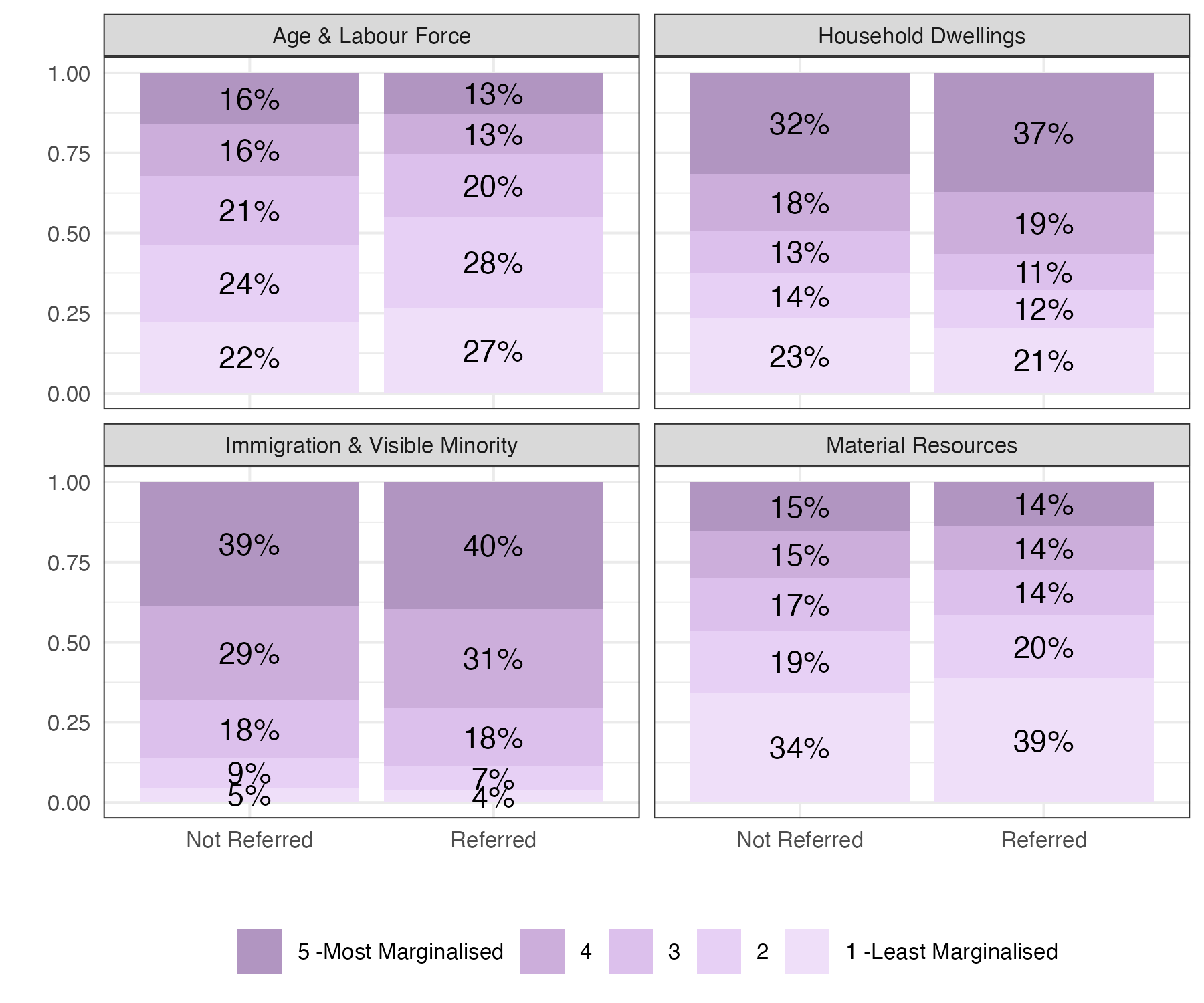
*

*Figure S4: Comparison of marginalization profiles between those referred and not referred to CRS*
